# Dr. James M. Showerman

**Published:** 1899-07

**Authors:** 


Dr. James M. Showerman, of Batavia, died at his residence in that village May 25, 1899, aged 59 years.

